# Can Fish and Shellfish Species from the Black Sea Supply Health Beneficial Amounts of Bioactive Fatty Acids?

**DOI:** 10.3390/biom11111661

**Published:** 2021-11-09

**Authors:** Albena Merdzhanova, Veselina Panayotova, Diana A. Dobreva, Katya Peycheva

**Affiliations:** Department of Chemistry, Faculty of Pharmacy, Medical University of Varna, 55 Marin Drinov Str., 9002 Varna, Bulgaria; veselina.ivanova@hotmail.com (V.P.); didobreva@gmail.com (D.A.D.); peytcheva@hotmail.com (K.P.)

**Keywords:** omega-3 fatty acids, seafood, nutritional quality, recommended daily intake, Black Sea

## Abstract

Fatty acids (FA) are among the most important natural biologically active compounds. A healthy diet involves the intake of different fatty acids especially from omega-3 (n-3) series. Seafood provides a very good source of polyunsaturated fatty acids (PUFAs), but in Bulgaria there is limited information regarding the n-3 PUFA contents in traditionally consumed seafood by the population. The aims of this study were to determine lipid content, omega-3 polyunsaturated fatty acids (EPA and DHA), and the recommended daily intake of eleven fish species, three bivalves, rapana, and shrimp harvested in the Western part of Black Sea, Bulgaria. Total lipids were extracted according to the method of Blight and Dyer and fatty acid composition was analyzed by GC/MS. Fatty acid profile showed differences among species. PUFA were found in high content among total lipids, especially in shellfish (60.67–68.9% of total lipids) compared to fish species (19.27–34.86% of total lipids). EPA was found in higher amounts in rapana (0.16 g/100 g ww) and two of pelagic species (up to 0.29 g/100 g ww), whereas DHA prevailed in demersal and the most of pelagic fish (0.16–1.92 g/100 g ww) and bivalves (0.16–1.92 g/100 g ww). The health beneficial n3/n6 and PUFA/SFA ratios were found in all analyzed species. The lower values of the lipid nutritional quality indices (AI < 1, TI < 1) and higher for h/H index (0.8–1.78 for fish and 1.52 to 4.67 for bivalves and shrimp) confirm that the commonly consumed Black Sea fish and shellfish may provide health benefits for local populations. This study shows the seafood amounts that can provide the minimum recommended intake of omega-3 polyunsaturated fatty acids.

## 1. Introduction

Currently, lots of studies are focused on natural functional foods that can provide both basic and essential nutrients for human health and thus reduce the risk of incidence of various chronic diseases [1,2,3,4,5,6,7]. Fatty acids (FA) are among the most important natural biologically active compounds. A number of FA especially omega-3 long chain polyunsaturated fatty acids (n-3 LC-PUFA), such as EPA and DHA, have been recognized as vital food components, due to the well-established relationship between their consumptions and cardiovascular diseases. Among the saturated, mono-, and PUFA groups, individual FAs have diverse effects on human health. While excessive intake of SFA and trans fatty acids is associated with negative effects on the human body [8,9], the n-3 long chain PUFA, such as eicosapentaenoic (C20:5 n-3, EPA) and docosahexaenoic (C22:6 n-3, DHA) acids, are linked with the decreased risk of cardiovascular diseases and other chronic non-communicable diseases (NCDs) [1,2,10,11]. The dietary sources of these key nutrients are scarce, because they are not found in terrestrial plants and the aquatic ecosystem is the most important producer and supplier of EPA and DHA [12]. The human body cannot effectively synthesize these FAs; therefore, the consumption of naturally rich foods is recommended. Marine lipids are excellent sources of EPA and DHA. A number of international health organizations [13,14,15] have advised regular seafood consumption due to their proven positive effects on human health. Various foods including seafood contain miscellaneous quantities of lipids and FAs. The availability of accurate information for the marine species with potential for being high-value sources of n-3 LC-PUFAs in different regions are scarce [15,16]. Moreover, the pattern of fatty acid consumption is specific to different geographical regions, cultures, and local traditions. National dietary guidelines include international recommendations for seafood consumptions without updating the national databases for nutrient composition of commonly consumed local fish and shellfish species. This results in inaccuracies when calculating the adequate seafood intakes for different regions to meet the international recommendations. Regular updates for the information about TL content and FA composition of commercially important marine species are key factors for assessment of the quality of marine lipids and the inclusion of local fish and shellfish species in healthy diets for prevention of chronic diseases. The Bulgarian population suffers from the deficiency of these nutrients due to the low consumption of seafood [11,17]. The existing national recommendations for health and nutrition advise regular fish consumption [10,11,13,18]. However, in Bulgaria there is limited information regarding the n-3 LCPUFA contents in seafood products, traditionally preferred and consumed by the local population. Earlier studies reported data on the lipid and fatty acid composition of some Black Sea fish and shellfish species and showed that total lipid content and FA profiles, including PUFA levels, are species-specific [19,20,21,22,23,24,25]. Having in mind these facts, the aims of this study were to determine lipid content, omega-3 polyunsaturated fatty acids (EPA and DHA), and the recommended daily serving of eleven fish species, three bivalve species, one gastropod, and one crustacean species harvested from the Western part of the Black Sea, Bulgaria, and regularly available on the Bulgarian fish markets. In addition, nutritional quality indices and ratios were employed for evaluation of the nutritional quality and potential health-beneficial significance of the species. This study reported data on the size of edible portion providing the recommended daily intake of omega-3 PUFAs.

## 2. Materials and Methods

### 2.1. Chemicals

All standard substances (Supelco 37 Component FAME Mix, PUFA No. 3 from Menhaden oil) were purchased from Merck (Darmstadt, Germany). All solvents (chloroform, methanol, and n-hexane) used in this study are of analytical grade (HPLC) and were purchased from Sigma–Aldrich (St. Louis, MO, USA). 

### 2.2. Sampling Procedures

Specimens of 11 fish species (*Sprattus sprattus*, *Engraulis encrasicolus ponticus*, *Trachurus mediterraneus ponticus*, *Alosa immaculata*, *Mugil cephallus*, *Sarda sarda*, *Belone belone*, *Pomatomus saltatrix*, *Neogobius melanostomus*, *Mullus barbatus*, *Scophthalmus maximus*), three bivalve species (*Mytilus galloprovincialis*, *Chamelea gallina*, *Donax trunculus*), one crustacean species (*Crangon crangon*), and one gastropod species (*Rapana venosa*) were purchased from local seafood retail markets (Varna, Bulgaria). The species were captured in the Bulgarian waters of the Black Sea during 2017 and 2018. The 16 species investigated including commercial designations (in Bulgarian), scientific names, biometric data, trophic groups, and trophic levels for the fish species are listed in Table 1. Individuals were put on ice and transported to the laboratory and immediately processed. Samples of each species with similar length were selected to ensure that any analytical differences were not size dependent.

Three individuals from each species *Alosa immaculata*, *Mugil cephallus*, *Sarda sarda,* and *Scophthalmus maximus* and ten individuals from *Belone belone*, *Pomatomus saltatrix*, *Neogobius melanostomus,* and *Mullus barbatus* were skinned and filleted. *Sprattus sprattus*, *Engraulis encrasicolus ponticus*, and *Trahurus mediterraneus ponticus* (15 individuals from each species) were eviscerated, the heads were removed, and processed with the skin. Each sample of shellfish was constituted of 20 individuals for bivalves, crustaceans, and gastropods. All tissues were cut into small pieces and homogenized with a laboratory blender (Isolab Laborgeräte GmbH Co., Eschau, Germany). All analyses were conducted in triplicate.

### 2.3. Fatty Acid Analysis

Total lipids (TL) were extracted according to the method of Bligh and Dyer [26]. Briefly, three grams of tissue homogenates were extracted sequentially with chloroform/methanol (1:2 *v*/*v*), chloroform/methanol (1:1 *v*/*v*), and chloroform with constant mixing for 30 min after each extraction step. Phase separation was achieved with NaCl solution in H_2_O (0.9% *w*/*v*). After centrifugation (3500× *g*, 15 min) bottom chloroform layer was collected with a Pasteur pipette, filtered through Na_2_SO_4_ and the solvent evaporated to dryness by rotary-evaporator. Total lipids were determined gravimetrically. The extracted lipids were diluted (1 mL) with hexane and stored in deep freeze at −18 °C prior further analysis. 

The fatty acids (FAs) of each species were determined as fatty acid methyl esters (FAME) after direct transmethylation with 2% sulfuric acid in methanol [27]. FAMEs were analyzed by gas chromatography using a Thermo Fisher Scientific FOCUS chromatograph (Waltham, MA, USA) equipped with a TRACE TR-5MS capillary column (30 m × 0.25 mm × 0.25 µm) and a PolarisQ ion trap mass spectrometer. The oven temperature was programmed from an initial oven temperature of 40 °C for 4 min, followed by a rate of 20 °C/min from 40 to 150 °C and raised from 150 to 235 °C at a rate of 5 °C/min, and then from 235 to 280 °C a rate of 10 °C/min for 5 min. Helium was used as the carrier gas at a flow rate of 1 mL/min. Fatty acid identification was performed by comparing their retention time and mass spectrum with MS spectra of the commercial FAME standards Supelco 37 and PUFA No. 3 from Menhaden oil under the same conditions of FAMEs. Individual FA were expressed both as percentage (%) of total amount of fatty acid. Percentages of total fatty acids data were converted to mg/100 g ww using a fatty acid conversion factor (XFA) for finfish, crustaceans, and mollusks, proposed by Weihrauch [28].

### 2.4. Nutritional Quality Indices (NQI)

The nutritional quality of studied fish and shellfish edible tissues was assessed by three nutritional indices—the atherogenicity index (AI), thrombogenicity index (TI) proposed by Ulbricht and Southgate [29], and the hypocholesterolemic to hypercholesterolemic ratio (h/H) according to Santos–Silva, Bessa, and Santos–Silva [30] as follows:

*Atherogenicity index (AI)*

$$AI=\frac{C12:0+\left(4\times C14:0\right)+C16:0}{\sum PUFA_{n-6}+\sum PUFA_{n-3}+\sum MUFA}$$

*Thrombogenicity index (TI)*

$$TI=\frac{C12:0+C14:0+C16:0}{\left(0.5\times PUFA_{n-6}\right)+\left(3\times PUFA_{n-3}\right)+\left(0.5\times MUFA\right)+\left(\frac{PUFA_{n-3}}{PUFA_{n-6}}\right)}$$

*Hypocholesterolemic to hypercholesterolemic ratio (h/H)*

$$h/H=\frac{C18:1n-9+C18:2n-6+C18:3n-3+C20:4n-6+C20:5n-3+C22:6n-3}{C14:0+C16:0}$$

### 2.5. Statistical Analysis

All analyses were performed in triplicate and the results were expressed as mean values ± standard deviation (SD). Data were tested for normality and mean values were compared by one-way ANOVA followed by a post-hoc Tukey’s test. Statistical significances between average amounts of FA groups among low, medium, and high fat fish species were considered at *p* ≤ 0.05 (Graph Pad Prism 6). Pearson correlation coefficientc were employed to assess the possible relationships between fatty acid profiles and trophic levels of fish species (STATISTICA 6.0). The corresponding trophic levels were obtained from www.fishbase.org (accesssed on 21 August 2021). Hierarchical clustering analysis was used to evaluate differences in the nutritional quality of studied fish and shellfish species based on the fatty acid profiles (SFA, MUFA, PUFA, n-3 PUFA, n-6 PUFA) and nutritional quality indices (n-6/n-3, PUFA/SFA, AI, TI, h/H) (STATISTICA 6.0). 

## 3. Results

### 3.1. Total Lipids and Fatty Acid Composition in Fish Species

Total lipid contents and fatty acid composition of the analyzed fish species are shown in Table 2.

#### 3.1.1. Total Lipids

Total lipid (TL) amounts showed significant differences (*p* < 0.05) between the eleven fish species. Among the planktivorous species, twice higher TL values are found in anchovy (*E. encrasicolus*) (10.05 g.100^−1^ g ww) compared to sprat (*S. sprattus*) (5.43 g.100^−1^ g ww). The most of carnivorous species present lower TL, in the range: from 2.11 g.100^−1^ g ww in round goby (*N. melanostomus*) to 6.82 g.100^−1^ g in ww horse mackerel (*T. mediterraneus*). Shad (*A. immaculata*), red mullet (*M. barbatus*), and bluefish (*P. saltatrix*) contain more than 13 g TL per 100 g ww. Based on the TL content, five of the analyzed fish—round goby, turbot, garfish, bonito, and gray mullet—can be classified as “low fat” (2–4% fat); sprat and horse mackerel as “medium fat” (4–8% fat); and anchovy, shad, red mullet, and bluefish as “high fat” (>8% fat) species [31,32]. Generally, the analyzed fish can be divided into two groups—with low and high lipid content.

#### 3.1.2. Fatty Acid Composition

**Figure 1:**
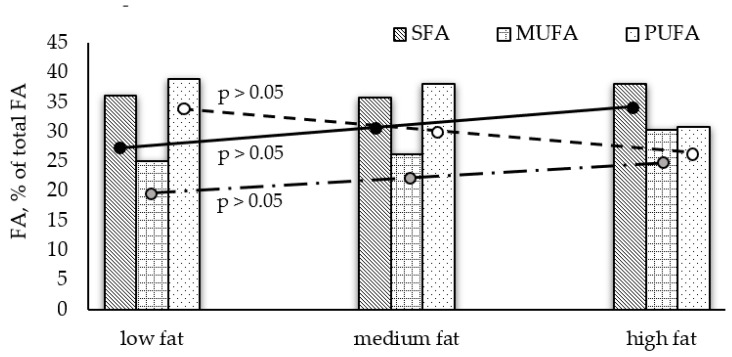
Differences in average fatty acid groups among the analyzed fish species with different lipid content.

In most of the analyzed fish, PUFA are the dominating group and a relative pattern PUFA > SFA > MUFA is determined; however, SFA prevail in red mullet, shad, and garfish. The highest amount of PUFAs are found in turbot (39.44%) and sprat (39.20%), and the lowest in red mullet (16.09%). Monounsaturated FA (MUFA) show significantly lower levels in all species. In our study, insignificant differences between average amounts of FA groups among low- and medium-fat fish (Graph Pad Prism 6, *p* > 0.05, Figure 1) were observed, whereas the high fat species contained significantly lower average PUFAs and higher SFAs levels.

It is known that marine fish are very good sources of PUFAs [4], which is confirmed by the presented results. When comparing the fatty acid profiles of species with varying lipid content, a negative relationship between TL and PUFA content was found in fish with TL < 5%; and a positive correlation between TL and SFA levels in high-fat fish (Figure 1). 

SFA was the major group in *M. barbatus* (49.13%), *A. immaculata* (37.44%), and *B. belone* (37.45%). The major saturated FA was palmitic acid (C16:0), which presented 15.50–30.10% of total FAs. The typical SFA profiles in most of the analyzed fish were palmitic (C16:0) > stearic (C18:0) > myristic (C14:0) acids, regardless of their TL content (Table 2). Only in *A. immaculata* did C14:0 have a higher level than C18:0.

Significantly larger variations in the profile of unsaturated FAs were found. The dominant MUFAs were palmitoleic (C16:1n-7) and oleic (C18:1n-9) acids. In most low-fat and high-fat fish species, C16:1 prevailed with the maximum of 21.90% of total FA, (*E. encrasicolus*), while in medium-fat species the major MUFA is C18:1n-9 (up to 17.90% of total FA, *M. cephallus*). In general, the individual MUFA levels were significantly different among fish species.

In the PUFA group, docosahexaenoic acid (DHA, C22:6n-3) showed the highest levels in nine of the fish species—within the following range: from 12.3% (horse mackerel, bonito) to 26.35% (turbot) of total FAs. In contrast, two of the species (red mullet and gray mullet) showed DHA values lower than 8% of total FAs. Other biologically active PUFAs found in most of the Black Sea fish species with values > 3% of total FAs were eicosapentaenoic acid (EPA, C20:5n-3), linoleic acid (LA, C18:2n-6), arachidonic acid (ARA, C20:4n-6), and alpha-linolenic (ALA, C18:3n-3) acid. The specific PUFA distribution of the most of studied fish was DHA > EPA > ARA, regardless of their TL content with the exception of red mullet and anchovy (DHA > ARA > EPA) and gray mullet (DHA > LA > ARA > EPA). In our study, DHA > EPA in all analyzed fish.

The sum of n-3 PUFAs was higher than the sum of n-6 PUFAs in nine of the eleven species (Table 3), with a maximum of 31.90% of total FAs (*S. maximus*). Despite that in red mullet and gray mullet n-6 prevailed, DHA is the major PUFA in these species. Most of the analyzed fish contained high EPA and DHA amounts—between 12.4% (*M. cephalous*) and 30.14% (*S. maximus*) of total FAs (Table 2). 

Due to the observed variations in the FA contents and composition, analyzed fish species may exert different influence on human health. The potential health benefits can be assessed through lipid quality ratios and indices, such as n-6/n-3, PUFA/SFA, AI, TI, and h/H (Table 3). In our study, n-6/n-3 ratios were in the range from 0.24 (*S. maximus*) to 1.31 (*M. barbatus*, *M. cephallus*) and for all analyzed species were below recommended values of 4.0 [33]. Variations were observed for PUFA/SFA ratios (Table 3), as *S. maximus* showed the highest (1.19), whereas red mullet showed the lowest PUFA/SFA ratio (0.35). Concerning the three nutritional indices, the results for AI and TI (AI > TI) were below 1.00 in most of the species, with the exception of the demersal red mullet (AI = 1.01). Moreover, h/H > 1.00, in range from 1.2 (anchovy) to 1.94 (round goby), except the bottom feeding red mullet (h/H = 0.82) (Table 3).

#### 3.1.3. Relationships between Trophic Levels and Fatty Acid Profiles in Fish 

**Figure 2:**
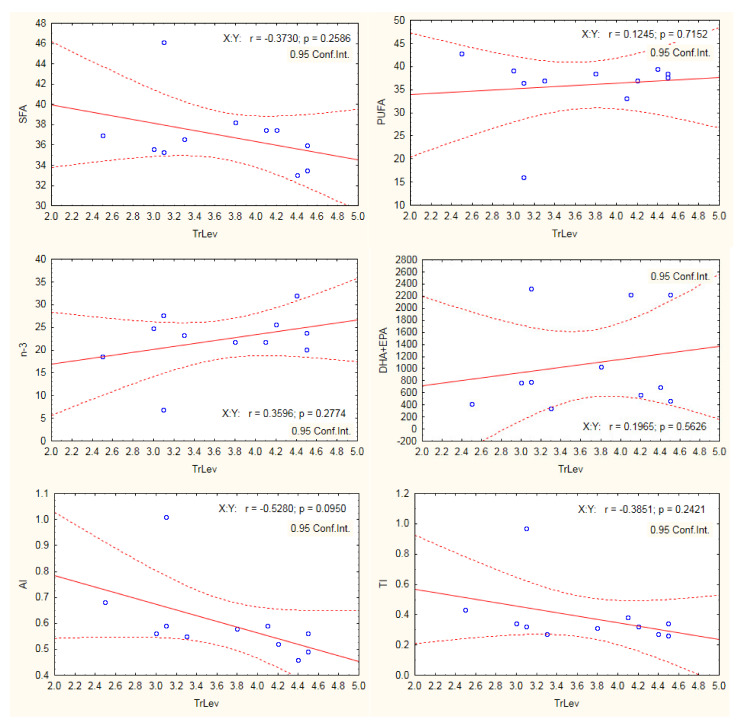
Pearson correlation between trophic levels (TrLev) and fatty acid groups and nutritional quality indices.

The Pearson correlation analysis was used to assess the potential relationship between trophic levels and fatty acid groups and nutritional quality indices of studied fish. Our results showed no significant effect of trophic levels on SFA, PUFA, n-3 PUFA, DHA + EPA, and TI (Figure 2). However, there was significant negative correlation between AI and the trophic levels. The low trophic level species were planktivorous species (with the exception of *M. cephallus* being omnivorous species) feeding mainly on phytoplankton. The phytoplankton is usually rich in PUFAs (both n-3 and n-6) with antiatherogenic potential.

### 3.2. Total Lipids and Fatty Acid Composition in Shellfish

Total lipid contents and fatty acid composition of the analyzed shellfish species are given in Table 4.

#### 3.2.1. Total Lipids

All studied shellfish species showed low TL levels lower than 2.60 g.100^−1^ g ww which is typical for these invertebrates and therefore are classified as “low-fat” food. 

#### 3.2.2. Fatty Acid Composition

**Figure 3:**
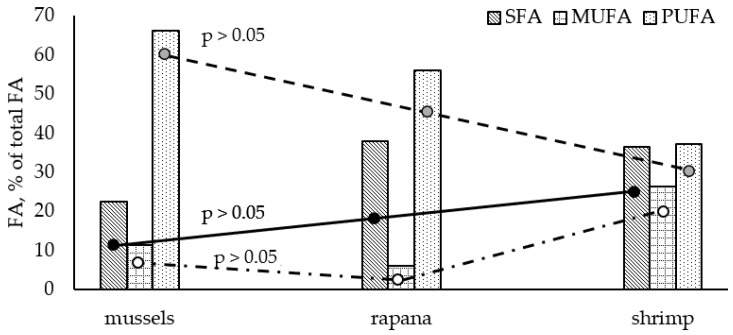
Differences in average fatty acid groups among the analyzed shellfish species.

In our study, PUFA prevailed in all shellfish species. A distinctive pattern of fatty acid groups was observed: PUFA > SFA > MUFA. Similar to the Black Sea fish species, the studied shellfish contained much lower levels of MUFA compared to the other two FA groups. The highest amount of PUFA was found in the white clams (*C. gallina* and *D. trunculus*), accounting for more than 68% of total FAs, followed by *M. galloprovincialis*, *R. venosa*, and *C. crangon* PUFA levels. In contrast to the analyzed fish, lower SFA values were found in the studied bivalves (from 20.3% in *C. gallina* to 25.49% in *M. galloprovincialis*). *R. venosa* and *C. crangon* contained significantly higher levels of SFA—up to 37.90% (*R. venosa*) of total FAs. Significant differences in the mean amounts of FA groups were observed among the studied shellfish (Graph Pad Prism 6, *p* > 0.05, Figure 3). *C. crangon* contained significantly lower PUFA and higher SFAs levels than bivalves. In general, compared to the Black Sea fish species, the shellfish were characterized by significantly higher PUFA and lower SFA levels. The observed SFA profile of studied bivalves and shrimp was similar to the results obtained for the fish species. Palmitic acid (C16:0) is the predominant FA, with the highest proportions found in *C. crangon* (27.38% of total FAs), followed by C18:0 and C14:0. Stearic acid (C18:0) is the major SFA in *R. venosa* (C18:0 > C16:0 > C14:0).

Among MUFAs, C16:1n-7 and C18:1n-9 are the major FAs. In the two white clams C18:1n-9 was higher than C16:1n-7 content, whereas in *M. galloprovincialis* and *C. crangon* C16:1n-7 prevailed. Different MUFA distribution was found for the carnivore *R. venosa*: C 20:1n-9 > C18:1n-9 ≥ C16:1n-7. 

Within the PUFA group, DHA and EPA are the predominant n-3 FAs and their sum accounts for approximately 38.55% of total FAs. *C. gallina*, *D. trunculus*, *M. Galloprovincialis,* and *C. crangon* contained higher DHA then EPA levels, within the range: from 14.75% (*C. crangon*) to 43.21% (*C. gallina*) of total FA were found in most of species. *R. venosa* contained two times higher amounts of EPA (29% of total FA or 173.7mg.100 g^−1^) than DHA. Therefore, *R. venosa* is an excellent source of this n-3 LCPUFA. The most abundant n-6 PUFAs in all species are ARA and LA (ARA > LA); however, *C. crangon* presented two times higher LA compared to ARA. Despite the observed variations in the unsaturated FA profiles, all species presented higher levels of n-3 PUFAs (23.4–55.26% of total FA) than n-6 PUFAs (8.64–23.59% of total FA).

In our study, all shellfish presented significantly lower n-6/n-3 ratios and AI and TI indices and higher PUFA/SFA (up to 3.40 for *C. gallina*) and h/H ratio (up to 4.67 for *Ch. gallina*) compared to the studied fish species (Table 5). Observed results for all shellfish species for n-6/n-3, AI and TI were below 1.00, while the h/H index presented significantly higher than 1.00 levels with lowest the value calculated for *C. crangon* (1.52).

European Food Safety Authority [13] recommends regular seafood consumption to provide a daily intake of 0.500 g EPA + DHA n-3 PUFAs. Table 6 presents the size of edible portions of analyzed species that provide the recommended daily amounts of EPA + DHA.

Considering these recommendations, the fish species *B. belone, S. sprattus, S. maximus*, and *M. barbatus* can be classified as “very good” sources, since they contain a 100 g edible portion supply between 570–780 mg of EPA + DHA. The other four species (*E. encrasicolus, T. mediterraneus, A. immaculata*, and *P. saltatrix*) presented the highest content of EPA + DHA in the range: 1000–2300 mg.100 g^−1^ ww and are therefore excellent sources of these n-3PUFAs. *N. melanostomus, S. sarda*, and *M. cephallus* contained lower EPA + DHA amounts (340–460 mg.100 g^−1^ ww). 

Taking into account the international recommendations, *C. gallina* and *M. galloprovincialis* can be classified as “very good” sources, since they contained a 100 g edible portion supplies between 750–900 mg of EPA + DHA, followed by *D. trunculus* (491.68 mg), whereas *R. venosa* and *C. crangon* contained significantly lower EPA + DHA amounts (in the range 240.1–253.1 mg.100 g^−1^ ww).

**Figure 4:**
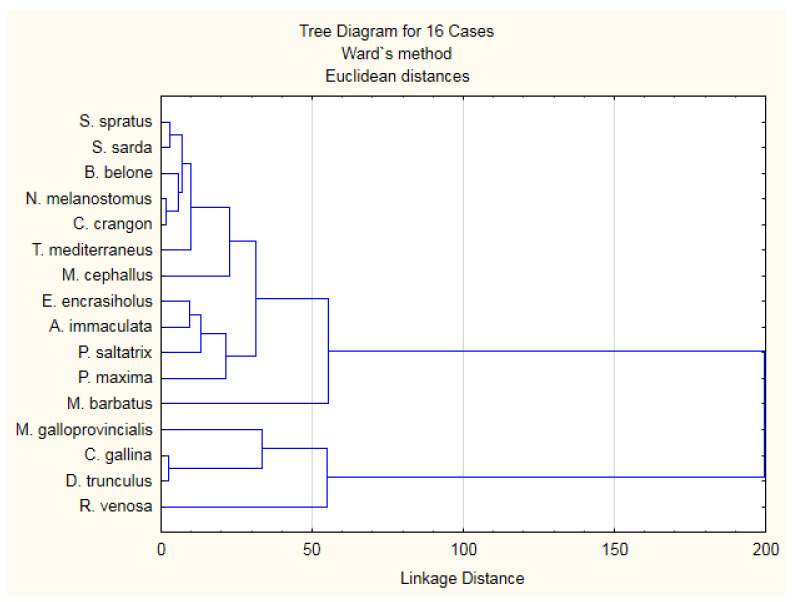
Hierarchical clustering analysis of the eleven fish and 5 shellfish species from the Black Sea.

Hierarchical clustering analysis was employed to evaluate differences in the nutritional quality of studied fish and shellfish based on the fatty acid profiles (SFA, MUFA, PUFA, n-3 PUFA, n-6 PUFA) and nutritional quality indices (n-6/n-3, PUFA/SFA, AI, TI, h/H) (Figure 4). 

Analyzed fish and shellfish species were clustered into four groups. Cluster 1 combines all fish species (except for *M. barbatus*) and *C. crangon*. Group 2 is *M. barbatus*. Cluster 3 contains the three bivalve species—*C. gallina*, *M. galloprovincialis*, and *D. trunculus*. Cluster 4 is for the gastropod *R. venosa*. The fish species from group 1 were characterized by higher lipid content, but species from group 3 were more desirable for human consumption in terms of beneficial lipids.

## 4. Discussion

### 4.1. Black Sea Fish Species

Considering the observed significant differences in lipid contents among the analyzed fish species and also between fish and shellfish confirms that this is one of the most variable and individual characteristics of fish. Our results differ from published data for the same species from the different regions of the Black Sea and other sea basins. Fluctuations in lipids among the seasons and locations reflect the biological adaptation of the organisms to the changes in the environment [12,34]. The commercial fishing in the Bulgarian Black Sea waters takes place from depths up to 100–120 m, where seawater has low temperature (reaches 7 °C in February). Pelagic and demersal species inhabiting these depths tend to accumulate lipids in order to overwinter at these low temperatures. TL content could be also used as an indicator of the nutritional and ecological status of the sea region because of the direct relationship between lipids and food availability [34,35]. Nikolsky [34] and Shulman [35] reported large fluctuations in TL content of *S. sprattus* and *E. encrasicolus ponticus* from the Crimean and the Bulgarian Black Sea coast. Kocatepe and Turan [20] presented higher TL content (13.90 g.100 g^−1^ ww) for anchovy and shad (*A. alosa,* 18.12 g.100 g^−1^ ww) from the Black Sea (Sinop region). Comparing the sprat TL content with our previous work [23] and for anchovy [34,35], comparable results are observed.

Higher TL (11.0 g.100 g^−1^ ww) were reported for horse mackerel (*T. mediterraneus*) from the Marmara Sea [36] and the Eastern Black Sea (Trabzon region), but similar for shad (12.0 g.100 g^−1^ ww) from the fish markets in Trabzon, [21], whereas significantly lower lipid levels (1.37–2.10 g.100 g^−1^ ww) were presented for same species from the Eastern Black Sea region [19] and the Southern Adriatic coast of Italy [37] compared to our results. 

Discrepancies between our results for demersal fish, such as red mullet (*M. barbatus*) and round goby (*N. melanostomus*), were found. Some authors [11,12] reported significantly lower TL values for the Black Sea *M. barbatus*, in the range 3.1–8.3 g.100 g^−1^ ww. In contrast, Brauer [34] presented very low TL amounts for *N. melanostomus* from the Western Baltic Sea (0.64–1.0 g.100 g^−1^ ww). In our previous work [15], comparable results for the Black Sea *N. melanostomus* were reported. There are limited data for the TL content of turbot (*S. maximus*) which is one of the most valuable fish species in all countries from the Black Sea region. Comparably lower results for TL (1.30 g.100 g^−1^ ww) were reported for turbot from the Black Sea [10] and from markets in China (1.15 g.100 g^−1^ ww) [35].

The pelagic garfish (*B. belone*) and bonito (*S. sarda*) are considered delicious and are preferable fish for consumptions in the Black Sea countries; however, there is limited data for their TL contents. Chuang et al. [21] reported significantly lower TL contents for garfish (0.2 g.100 g^−1^ ww) and bonito (0.5 g. 100 g^−1^ ww) and significantly higher TL (5.8 g.100 g^−1^ ww) for gray mullet (*M. cephallus*). Kocatepe and Turan [20] found similar TL amounts for garfish (3.31 g.100 g^−1^ ww) and high-fat bluefish (12.3 g.100 g^−1^ ww). In contrast, Tufan et al. [38] reported three times higher TL content (8.3–11.5 g.100 g^−1^ ww) for garfish from the Eastern Black Sea.

For the assessment of the potential of the Black Sea fish species as valuable sources of bioactive FAs, knowing the detailed FA profiles with an emphasis on n-3 LC-PUFA is of crucial importance. This study provides up to date information for both profiles (percentage composition) and content (absolute amounts) of FA in eleven commercially important fish species from the Bulgarian part of the Black Sea. 

The main result in our study is that the most of analyzed fish species are very good sources of polyunsaturated FAs and can supply these bioactive components. As mentioned above, species with low TL contents (<4 g.100 g^−1^ ww) show PUFA-rich profiles, while medium- and high-fat fish contain higher absolute amounts (as mg.100 g^−1^ ww). However, the lipid fractions of two of the “high-fat” species—red mullet (*M. barbatus*) and shad (*A. immaculata)*—are characterized by the dominance of SFA group. Although increased SFA consumption is associated with an increased risk for the development of cardiovascular diseases [8,9,16], the studied species contained significantly lower saturated FA levels compared to other meat products, but not all SFAs may have harmful effects on CVD. According to FAO/WHO [10] and Praagman et al. [39], the individual SFAs with atherogenic and thrombogenic potential are lauric (C12:0) and myristic (C14:0) acids. These two fatty acids were minor components (<3–4% of total FAs) in the analyzed fish species. Compared to other studies, the observed SFA levels are similar to those reported by Ozogul et al. [19], Kocatepe and Turan [20], and Chuang et al. [21] for anchovy, bluefish, turbot, garfish, and goby from the Black Sea, but lower than horse mackerel from the Marmara Sea [36] and black goby from the Ionian Sea [40]. 

According to Prato et al. [16], MUFAs are “beneficial” for human health due to their properties to decrease the low-density lipoproteins (LDL) and blood cholesterol levels. Moreover, oleic acid (C18:1 n-9) participates in the stimulation of bile secretion which is important for the optimal nutrient absorption [41], whereas palmitoleic acid (C16:1 n-7) may influence positively the functions of immune cells [42]. Among MUFAs, C16:1 n-7 and C18:1 n-9 are the major FAs found in the Black Sea fish, with significant species-specific variation. In demersal species (goby and turbot) C16:1 n-7 prevail, while red mullet contains almost equal levels of C16:1 n-7 and C18:1 n-9 (C18:1 n-9 ≥ C16:1 n-7). Oleic acid prevails in sprat, horse mackerel, bonito, and gray mullet, while C16:1 n-7 dominated in the lipid fraction of anchovy, shad, bluefish, and garfish. No correlation was found between C16:1 n-7 and C18:1 n-9 and TL contents in fish. Significantly higher MUFA levels (48.5% of total FAs) are reported for Black Sea shad from Trabzon region [21], whereas Kocatepe and Turan [20] presented similar MUFA contents (30.26% of total FAs) for this species from the Sinop region. Ozden et al. [36] found comparable MUFA levels in horse mackerel from the Marmara Sea, while Chuang et al. [21] found higher levels (37.5%) for the same species from the Black Sea. Merdzhanova and Dobreva [24] reported similar data for Black Sea round goby MUFAs content, whereas Brauer et al. [43] reported significantly lower MUFAs (average 14.6% of total FAs) for round goby. Our results differ from those reported by Kocatepe and Turan [20] for Black Sea anchovy MUFA (only 19.5%) and are close to garfish and bluefish MUFA levels. Chuang et al. [21] reported lower MUFA levels for garfish (16%) and gray mullet (22.2%), higher for bonito (31%), and similar for red mullet from the Black Sea. 

Significant variations in the levels of total and individual PUFA were found for the studied Black Sea fish. Ten PUFAs are identified with DHA being the major FA in all species. DHA contents varied widely in the following range: from 4.68% of total FAs (*M. barbatus*) to 26.35% of total FAs (*S. maximus*). The second most abundant FA was EPA, followed by ARA (arachidonic acid, C20:4n6), LA (linoleic acid, C18:2n6), and ALA (alpha-linolenic acid (C18:3 n3). According to many studies, the marine fish lipids contain significantly higher n-3 than n-6 PUFA levels [16,19,20,21,32,44,45]. In this study the sum of the most abundant n-3 PUFA (EPA and DHA) accounts from 36% of total PUFA (*M. barbatus*) to 76.4% of total PUFA (*S. maximus*). One possible explanation for the observed high contents of both n-3 LCPUFAs is the fact that plankton (phyto- and zoo-) is a major part of the fish diet, regardless of the species. According to Prato et al. [40] and Gladishev et al. [12], plankton lipids are the richest sources of n-3 PUFA (especially the long chain EPA and DHA) in aquatic food web. We found that the low-fat species contained higher DHA proportions compared to high fat species. Huynh and Kitts [46] reported similar results for sardine, hake, herring, and pink salmon from the Pacific Northwest region and also found that low-fat species contained higher DHA compared to high-fat fish. In this study, the levels of EPA were always lower than levels of DHA, similarly to the results obtained by Prato et al. [16] for seafood from the Ionian Sea. The DHA proportions in shad and bonito were higher, whereas those for horse mackerel, gray mullet, and garfish were lower compared to the results of Chuang et al. [21], and similar to those reported by Kocatepe and Turan [20] for anchovy, bluefish, and red mullet. 

The reported results (Table 6) demonstrated significant variations in EPA + DHA contents and thus different contribution of each of the analyzed species in EPA + DHA dietary needs. In contrast to our results, significantly lower amounts for EPA + DHA mg.100 g^−1^ ww were reported for horse mackerel from the Marmara Sea (793 mg) [36], but higher values for Ionian Sea red mullet (1086 mg) [16]. Kocatepe and Turan [20] presented considerably lower levels for red mullet (150 mg), anchovy (250 mg), shad (210 mg), bluefish (140 mg), and garfish (290 mg). Chuang et al. [21] reported very low EPA + DHA amounts for Black Sea shad (55 mg) and gray mullet (54 mg), but similar for horse mackerel (750 mg). The observed differences in species from different regions of the Black Sea indicate the need for detailed study of FA contents of local species in order to obtain reliable information.

The low consumption of fish and shellfish and the lack of data on FA composition of local species are major hindrances toward healthy choices of the Bulgarian consumers. The modern dietary pattern in western societies is characterized by higher SFA and n-6 PUFA and lower n-3 PUFA consumption and the replacement of n-3 PUFA with n-6 in the western population could be related to the increased risk of obesity and CVD [3,40,47]. The balanced FA profile could play an important role in the prevention of various CVD [3]. Two important ratios with recommended ranges: for n-6/n-3 ratio—0.45–4.0 [47] and PUFA/SFA > 1.0 [3,47] are used for lipid quality assessments. In our study, the n-6/n-3 levels varied significantly among species but fell within the recommended range. Favorable results were found for PUFA/SFA ratios, as nine of the studied fish species showed values ≥ 1.0. Only in red mullet and shad (TL ≥ 13 g.100 g^−1^ ww) was the PUFA/SFA ration <1.0. Kocatepe and Turan [20] and Chuang et al. [21] presented lower values for both ratios for Black Sea anchovy, bluefish, shad, horse mackerel, gray mullet, red mullet, garfish, and bonito. Other studies reported similar n-6/n-3 and PUFA/SFA ratios for Black Sea horse mackerel, gray mullet, and turbot [19] and for mackerel, jack mackerel, Chilean hake, and croaker from the South Pacific [45]. Based on the reported results, we can conclude that all studied Black Sea fish have well-balanced and beneficial FA profiles regardless of the species or TL content.

Indices of atherogenicity (AI), thrombogenicity (TI), and hypocholesterolemic/hypercholesterolemic ratio (h/H) were used to assess the functional potential of fish lipids to stimulate platelet aggregation. The recommended limit for AI and TI was < 1.0 [29], whereas for h/H index: >1.0 [30] which can be used for prediction of the potential of FA composition to protect against cardiovascular diseases development. In this study, all of the analyzed species showed AI < 1.0 (0.46–1.01) and TI < 1.0 (0.26–0.97) values and h/H index from 0.82 to 1.94. Prato et al. [16] reported similar results for AI, TI and h/H indices of *M. barbatus* and *S. maximus* from the Ionian Sea. Rincón–Cervera et al. [45] found significantly higher h/H levels (>2) for South Pacific fish species. Gonçalves et al. [48] showed lower levels for AI and TI, but higher for h/H index for fourteen marine fish from the Brazilian north-eastern coast. In contrast, Zhang et al. [32] presented higher AI and TI, but lower h/H levels for 22 marine fish species from Pearl River estuarine, China. Therefore, the obtained low AI and TI and high h/H values describe Black Sea fish as healthy food with very good anti-atherogenic, anti-thrombogenic, and hypocholesterolemic potential.

### 4.2. Black Sea Shellfish

In this study, the total lipid amounts showed minimal differences between bivalve and shrimp species, with the lowest values in *R. venosa* (0.81 g.100 g^−1^ ww.). There is a lack of information of TL and fatty acid composition for most of the analyzed shellfish from the Black Sea. Therefore, we compared our results with same shellfish species from different regions of the Black Sea and other sea basins. Similar TL content was reported for *M. galloprovincialis* from the southern part of the Black Sea [49], whereas Prato et al. [50,51] found lower TL for *M. galloprovincialis* from the Ionian Sea. Ozden et al. [52] presented significantly lower TL amount for *C. gallina* (0.9 g.100 g^−1^ ww) and *D. trunculus* (0.8 g.100 g^−1^ ww) from the Northern Marmara Sea, whereas Calakoglu et al. [53] showed comparable TL for *C. gallina* from the Southern Marmara Sea. Orban et al. [54] obtained lower TL amounts for *C. gallina* from the Adriatic Sea. Comparably low TL contents were found for the Black Sea *R. venosa* [55], and *R. venosa* from Qingdao seafood markets in China [56]. In our previous studies, *R. venosa* presented higher TL content (1.26 g.100 g^−1^ ww) [22] and lower TL values (0.5 g.100 g^−1^ ww) [57]. Information on *C. crangon* lipid composition is limited. Turan et al. [58] reported lower TL (0.95 g.100^−1^ g ww) for *C. crangon* from the Black Sea (Sinop), whereas Merdzhanova et al. [59] presented comparable levels (1.35 g.100^−1^ g ww). Based on the reported results, we can conclude that shellfish from the Bulgarian Black Sea coast have a health beneficial TL content compared to other data. The Black Sea has specific environmental factors, such as abundant nutritional substance supplies by river large inputs which results in high plankton density [53].

The summary of the data observed in this study showed that all invertebrate species contain higher average PUFA (58.31% of total FA) and lower average SFA levels (28.3% of total FA) compared to the fish species analyzed. 

Prato et al. [16] and Biandolino et al. [51] found significantly higher SFA levels (more than 40% of total FA) for *M. galloprovincialis*. Higher SFA values were reported for *C. gallina* [54] and *D. trunculus* [60]. The determined SFA levels for *R. venosa* were lower compared to those presented by Popova et al. [55], and higher than data reported by Turan et al. [58] for *C. crangon.* Among SFA, both unfavorable C12:0 and C14:0 acids were found in minor contents. In all bivalve species, C12:0 was not detected and C14:0 was found in trace amounts. The relatively higher amounts of C12:0 (<1.0% of total FA, 0.0–8.5 mg.100 g^−1^ ww) and C14:0 (1–2.6% of total FAs, 15–36 mg.100 g^−1^ ww) in *R. venosa* and *C. crangon* did not affect the quality of their lipids. 

In all studied species the group of MUFA was found in the lowest levels. Higher MUFA amounts were reported in the literature for *M. galloprovincialis* [16,51], *C. gallina* [54], and *D. trunculus* [60], compared to our results. Similar MUFA values were determined for *R. venosa* [55] and *C. crangon* [58]. In general, the two main monounsaturated FAs C16:1n-7 and C 18:1n-9 accounted on average 90% of total MUFA, but their distribution differed among shellfish species. Herbivorous bivalves contained higher C16:1n-7 and C18:1n-9 amounts than the carnivorous *R. venosa*, whereas the omnivorous shrimps showed the richest and most diverse MUFA profile. *M. galloprovincialis* and *C. crangon* contained higher C16:1n-7 than C18:1n-9. Similar levels for C18:1 n-9 and C16:1 n-7 were reported by Prato et al. [16] and Biandolino et al. [51] for *M. galloprovincialis* and *C. crangon* from the Black Sea [58]. Popova et al. [55] reported that C16:1 n-7 > C18:1 n-9 in *R. venosa*, which differed from our results (C16:1 n-7 ≅ C18:1 n-9). Discrepancy with our results were found for *C. gallina*—C16:1 n-7 < C18:1 n-9 [54], but agreement for *D. trunculus* [60]. Observed differences in MUFA contents may be explained with specific environmental factors (biotic and abiotic) [58].

In shellfish, DHA prevailed in the PUFA group (with the exception of *R. venosa*). PUFA profiles differ among species: the three bivalves’ FA pattern is: DHA > ARA > DPA > EPA; *R. venosa*: EPA >> DHA > DPA > ARA; and *C. crangon*: DHA > LA > EPA > ARA. The sum of the four fatty acids accounts for 85–90% of total PUFAs. Bivalves have low capacity to synthesize highly unsaturated PUFAs; consequently, their FA profiles are strongly influenced by the food availability and its abundance in the region [25]. Both n-3 PUFAs are specific markers for zooplankton and dinoflagellates (DHA) and diatoms (EPA), which synthesize them in high quantities. EPA and DHA proportions varied significantly among species, but the observed results are comparable with literature data for *M. galloprovincialis* from the Black Sea [25], *C. crangon* [58], *R. venosa* [55], *C. gallina* [54], and for *D. trunculus* [60]. In contrast, Prato et al. [16] and Biandolino et al. [51] reported lower values for DHA (10.6% of total FAs) and higher for EPA (12% of total FAs) for *M. galloprovincialis*. Compared to the Black Sea fish analyzed, docosapentaenoic acid (DPA, C22:5n-3) in most of the invertebrate species (except for *C. crangon*) was detected (in the range 1.57–6.93% of total FAs or 34.3–109.2 mg.100 g^−1^ ww). DPA is an interesting n-3 LCPUFA which relative deficiency is associated with atherosclerosis, coronary thrombosis, and other coronary diseases [7].

The most specific characteristic of the Black Sea shellfish was the predominance of n-3 PUFA with focus on EPA and DHA contents. In our study, the sum of EPA + DHA varied significantly among analyzed specimens which can supply from 48% to 180% of recommended daily intake (RDI). There is limited information for the EPA + DHA absolute amounts in the most of the analyzed Black Sea species. The Mediterranean mussel (*M. galloprovincialis*) is one of the most studied species. Compared to our results, Prato et al. [27] reported two times lower EPA + DHA contents (320.79 mg.100 g^−1^ ww) for Ionian Sea mussels and Panayotova et al. [25], and lower average amounts (410 mg.100 g^−1^ ww) for the same Black Sea species. In our earlier study, data on raw and cooked *R. venosa* were presented [22]. DHA was not detected in *R. venosa* lipids, while EPA (146 mg.100 g^−1^ ww) and DPA (34 mg.100 g^−1^ ww) contents were lower compared to present results. No comparable information about EPA and DHA contents was found in the literature for the other species (*C. gallina, D. trunculus*, and *C. crangon*) from different parts of the Black Sea. These are the new results for EPA + DHA contents of studied Black Sea shellfish species. Based on the calculated results, in order to supply 500 mg of these long-chain FAs, it is necessary to consume portions between 55.6 and 198 g depending on the species (Table 6).

All studied shellfish contained higher n-3 than n-6 PUFAs, which results in health beneficial values (Table 5) for n-6/n-3 and PUF/SFA ratios and nutritional quality indices (AI, TI, h/H). We found significant differences between n-6/n-3 and PUFA/SFA ratios presented in literature for the same species. Lower values for n-6/n-3, PUFA/SFA and h/H ratios were reported for Ionian Sea *M. galloprovincialis* [16], but higher for AI and TI in *M. galloprovincialis* [16], *C. crangon* [58], *R. venosa* [55], *C. gallina* [54], and *D. trunculus* [60]. Similar results for all indices and ratios were reported in our previous study of Black Sea *M. galloprovincialis* [25] and for h/H values in *R. venosa* [55]. In all studied Black Sea shellfish species, we found low n-6/n-3 and high PUFA/SFA ratios. The average AI and TI indices were <0.52 (AI) and <0.36 (TI), while h/H ratios were up to 4.6 and were within the recommended diapasons. Consequently, these species were also promising and valuable natural sources of essential biologically active fatty acids besides Black Sea fish.

### 4.3. Nutrition Importance for the Bulgarian Population

According to WHO [11] the main cause of morbidity in Bulgaria is from CVD. Low seafood consumption in Bulgaria is related to low omega-3 PUFA intake which results in health problems in all age groups. The existing national normative documents related to the health and nutrition of the Bulgarian population recommend increase of seafood intake [18]. Unfortunately, no detailed information and requirements for the intake of specific biologically active FAs has been provided. Moreover, there is insufficient information on the FA profiles of traditionally consumed local species. It is difficult for dietitians to make the right choice among the affordable fish and shellfish that can provide the recommended or higher amounts of n-3 LCPUFAs. Our study presents new data that will be increase the knowledge for the nutritional quality of seafood commonly consumed in Bulgaria. In summary, the Black Sea fish and shellfish show very diverse fatty acid profiles. A number of unsaturated biologically active fatty acids were identified. The main interest was focused on the quantities of EPA + DHA in local species available on the Bulgarian fish markets. Eight of the studied fish species contained significantly higher than recommended 500 mg EPA + DHA per 100 g edible portion, which made them excellence sources of these fatty acids. Moreover, a 100-g portion of *E. encrasicolus* and *P. saltatrix* supplied more than 2000 mg EPA + DHA. Among shellfish species, 100 g of *C. gallina* and *M. galloprovincialis* may provide beneficial levels (>750 mg) of EPA and DHA.

## 5. Conclusions

This research increases the knowledge of FA profiles of commercially important fish and shellfish species, traditionally consumed in the Black Sea region. Most of the studied species are valuable food sources for humans regarding their EPA + DHA contents and may supply on average: 1254.6 mg n-3 LC-PUFA per 100 g ww. The pelagic species contained higher n-3 amounts compared to the demersal ones. The low-fat demersal *N. melanostomus* and *S. maximus* lipids comprised significantly high EPA + DHA contents, 340–670 mg.100 g^−1^ ww. *E. encrasicolus* proved to be the richest source of EPA + DHA (2328.7 mg.100 g^−1^) that could contribute to 465.7% of the recommended intake. Other valuable species—*A. immaculata*, *P. saltatrix*, and *T. mediterraneus*—can supply between 1030 and 2220 mg EPA + DHA per 100 g. Studied shellfish contain on average 596.12 mg n-3 LCPUFA per 100 g ww, as striped venus clam *C. gallina* appears to be an excellence source of EPA + DHA, and *R. venosa* contains the highest EPA amounts. No significant effect of trophic levels on SFA, PUFA, n-3 PUFA, DHA + EPA, and TI was found; however, a significant negative correlation between AI and trophic levels was observed. This study demonstrates that Black Sea fish are abundant and sustainable sources of n-3 LCPUFAs when following the recommendations for consumption levels for specific health benefits.

## Figures and Tables

**Table 1:** Scientific names, commercial designations (in Bulgarian), biometric data (mean ± SD), trophic groups, and trophic levels (for the fish species).

Species	Common Name	Commercial Designation ^1^	Weight, g	Length, cm	Trophic Group	Trophic Level
***Pelagic fish species***						
*Sprattus sprattus* (Linnaeus, 1758), Family Clupeidae	Sprat	Тpицoна	12.0 ± 1.5	11.5 ± 0.5	Planktivorous	3.0 ± 0.07
*Engraulis encrasicolus ponticus* (Linnaeus, 1758), Family Engraulidae	Anchovy	Aншoа	23.0 ± 2.0	16.0 ± 0.5	Planktivorous	3.1 ± 0.36
*Trahurus mediterraneus* (Steindachner, 1758), Family Carangidae	Horse mackerel	Сафpид	22.0 ± 1.5	15.0 ± 1.0	Carnivorous	3.8 ± 0.3
*Alosa immaculata* (Bennett, 1835), Family Clupeidae	Shad	Каpагьoз	320.0 ± 15.0	28.0 ± 2.0	Carnivorous	4.1 ± 0.58
*Mugil cephallus* (Linnaeus, 1758), Family Mugilidae	Gray mullet	Кефал	290.0 ± 10.0	35.0 ± 2.5	Planktivorous, Carnivorous Detritivorous (or omnivorous)	2.5 ± 0.17
*Sarda sarda* (Bloch, 1793), Family Scombridae	Atlantic bonito	Паламуд	420.0 ± 15.0	40.0 ± 2.0	Carnivorous	4.5 ± 0.00
*Belone belone* (Linnaeus, 1761), Family Belonidae	Garfish	Заpган	55.0 ± 4.0	35.0 ± 2.0	Carnivorous	4.2 ± 0.40
*Pomatomus saltatrix* (Linnaeus,1758), Family Pomatomidae	Bluefish	Чеpнoкoп	60.0 ± 5.0	18.5 ± 1.5	Carnivorous	4.5 ± 0.30
***Demersal fish species***						
*Neogobius melanostomus* (Pallas, 1814), Family Gobiidae	Goby	Кая	69.5 ± 5.0	17.0 ± 1.5	Carnivorous	3.3 ± 0.1
*Mullus barbatus* (Linnaeus, 1758), Family Mullidae	Red mullet	Баpбуна	40.0 ± 5.0	16.0 ± 1.5	Carnivorous	3.1 ± 0.1
*Scophthalmus maximus* (Linnaeus, 1758), Family Scophthalmidae	Turbot	Калкан	1400 ± 30.0	45.0 ± 3.0	Carnivorous	4.4 ± 0.0
***Shellfish***						
*Mytilus galloprovincialis*, Family Mytilidae	Mediterranean mussel	Чеpна мида	0.85 ± 0.13 ^2^	4.2 ± 1.1	Herbivorous	NA
*Chamelea gallina*, Family Veneridae	Striped venus clam	Бяла мида	0.34 ± 0.32 ^2^	2.4 ± 0.6	Herbivorous	NA
*Donax trunculus*, Family Donacidae	Wedge clam	Бяла мида	0.51 ± 0.57 ^2^	3.1 ± 0.9	Herbivorous	NA
*Rapana venosa*, Family Muricidae	Veined rapa whelk	Рапан	25.5 ± 2.5 ^2^	6.27 ± 0.9	Carnivorous	NA
*Crangon crangon*, Family Crangonidae	Common shrimp	Скаpида	0.25 ± 0.05 ^2^	1.5 ± 0.2	Omnivorous	NA

^1^ in Bulgarian. ^2^ soft tissue weight without shells.

**Table 2:** Total lipids (g.100^−1^ g wet weight) and fatty acid composition (mg.100^−1^ g), in the brackets (% of total FAs) of 11 fish species from the Black Sea.

	*Spratus* *spratus*	*Engraulis encrasiholus ponticus*	*Trahurus mediterraneus ponticus*		*Alosa* *immaculata*	*Mugil* *cephallus*
Total lipids (g.100^−1^ g ww)	5.40 ± 0.30	10.00 ± 0.80	6.80 ± 0.30		13.00 ± 0.70	3.80 ± 0.32
FA content (mg.100^−1^ g)						
C12:0	81.46 ± 20.53 (2.96)	49.34 ± 6.43 (0.53)	162.19 ± 36.20 (2.61)		94.68 ± 20.51 (0.79)	36.28 ± 4.25 (1.07)
C14:0	86.41 ± 17.65 (3.14)	171.12 ± 18.61 (1.85)	261.76 ± 25.10 (4.20)		325.83 ± 30.10 (2.72)	91.39 ± 8.10 (2.69)
C16:0	427.08 ± 35.42 (15.35)	2775.62 ± 78.52 (29.97)	1025.58 ± 42.35 (14.49)		3057.58 ± 57.63 (25.21)	94.85 ± 9.23 (28.65)
C18:0	121.97 ± 27.83 (4.44)	115.57 ± 8.55 (1.25)	379.08 ± 21.43 (6.08)		499.12 ± 38.65 (4.16)	75.96 ± 5.15 (2.23)
C20:0	75.12 ± 20.15 (2.37)	54.56 ± 3.70 (0.59)	221.37 ± 16.22 (3.55)		157.91 ± 12.70 (1.32)	27.63 ± 1.34 (0.81)
C22:0	92.05 ± 18.55 (3.35)	37.01 ± 2.04 (0.40)	144.45 ± 10.54 (2.32)		185.72 ± 9.54 (1.55)	22.88 ± 1.07 (0.67)
C24:0	59.03 ± 12.58 (2.15)	32.69 ± 2.23 (0.35)	141.28 ± 11.00 (2.27)		145.26 ± 11.30 (1.21)	17.68 ± 0.46 (0.52)
SFA	977.45 ± 65.15 (35.55)	3265.84± 110.15 (35.26)	2374.79 ± 97.50 (28.18)		4488.0 ± 62.46 (37.44)	1256.74 ± 60.37 (36.94)
C14:1	34.92 ± 5.63 (1.27)	20.03 ± 1.56 (0.22)	37.98 ± 4.32 (0.61)		83.94 ± 21.50 (0.70)	15.12 ± 0.38 (0.44)
C16:1	175.92 ± 25.14 (6.49)	2028.77 ± 62.17 (21.91)	429.26 ± 28.50 (6.90)		1479.06 ± 52.40 (12.34)	251.31 ± 18.33 (7.39)
c-C18:1n-9	227.63 ± 20.30 (8.28)	457.08± 33.71(4.94)	567.01 ± 45.10 (9.12)		1199.86 ± 46.15 (10.01)	609.39 ± 47.51 (17.91)
C20:1	77.81 ± 8.72 (2.83)	35.13 ± 2.83 (0.38)	101.75 ± 10.55 (1.64)		272.79 ± 16.84 (2.28)	23.59 ± 1.55 (0.69)
C21:1	96.44 ± 10.41 (3.51)	44.74 ± 2.04 (0.48)	156.44 ± 14.23 (2.52)		300.75 ± 20.36 (2.51)	27.06 ± 2.03 (0.80)
C24:1	81.43± 7.55(2.96)	29.92 ± 7.25 (0.32)	162.15 ± 12.30 (2.61)		188.47 ± 15.46 (1.57)	20.02 ± 0.85 (0.59)
MUFA	694.15 ± 55.30 (25.25)	2615.67 ± 31.50 (28.24)	1454.60 ± 64.70 (29.39)		3524.88 ± 13.80 (29.41)	946.49 ± 46.88 (27.82)
C18:3n-6	50.73 ± 4.69 (1.85)	20.98 ± 0.07 (0.23)	110.05 ± 7.46 (1.77)		101.47 ± 8.65 (0.85)	53.25 ± 3.08 (1.57)
C18:2n-6	108.15 ± 9.27 (3.93)	323.94 ± 18.22 (3.50)	337.14 ± 15.80 (5.42)		684.24 ± 36.50 (5.71)	251.31 ± 25.70 (7.39)
C18:3n-3	99.55 ± 10.23 (3.62)	36.35 ± 5.30 (0.39)	219.49 ± 10.45 (3.53)		225.85 ± 10.87 (1.88)	61.96 ± 5.67 (1.82)
C20:3n-3	89.52 ± 6.43 (3.26)	197.18 ± 12.35 (2.13)	95.83 ± 6.33 (1.54)		160.52 ± 7.45 (1.34)	111.80 ± 9.05 (3.29)
C20:3n-6	54.53 ± 4.25 (1.98)	Nd	54.89 ± 3.25 (0.88)		101.43 ± 3.58 (0.85)	95.55 ± 8.10 (2.81)
C20:2n-6	51.77 ± 5.10 (1.88)	44.41 ± 4.10 (0.48)	197.58 ± 20.05 (3.18)		116.35 ± 3.10 (0.97)	41.01 ± 2.38 (1.21)
C20:4n-6	84.10 ± 9.15 (3.06)	377.98 ± 28.64 (4.08)	219.33 ± 18.14 (3.53)		229.93 ± 20.45 (1.92)	155.57 ± 12.20 (4.57)
C20:5n-3	122.72 ± 11.04 (4.46)	367.31 ± 20.17 (3.97)	264.03 ± 15.38 (4.24)		246.20 ± 17.40 (2.05)	112.32 ± 8.04 (3.30)
C22:6n-3	368.79 ± 31.18 (13.41)	1961.38 ± 45.80 (21.18)	771.02 ± 42.60 (12.40)		1982.73 ± 40.39 (16.54)	272.80 ± 18.15 (8.02)
C22:2n-9	47.58 ± 3.49 (1.74)	50.58 ± 3.78 (0.55)	121.36 ± 9.37 (1.95)		124.40 ± 10.10 (1.04)	43.59 ± 4.08 (1.28)
PUFA	1077.70 ± 23.00 (39.20)	3380.13 ± 41.55 (36.50)	2390 ± 60.15 (38.44)		3973.12 ± 76.26 (33.15)	1199.17 ± 25.75 (35.24)
	***Sarda*** ***sarda***	***Belone*** ***belone***	***Pomatomus*** ***saltatrix***	***Neogobius melanostomus***	***Mullus*** ***barbatus***	***Scophthalmus maximus***
Total lipids (g.100^−1^ g ww)	3.10 ± 0.28	3.00 ± 0.25	15.40 ± 1.05	2.10 ± 0.15	14.70 ± 0.60	2.60 ± 0.20
FA content (mg.100^−1^ g)						
C12:0	82.48 ± 5.36 (3.00)	41.81 ± 3.70 (1.57)	186.88 ± 20.15 (1.31)	55.09 ± 1.40 (3.02)	143.73 ± 1.40 (1.06)	12.83 ± 0.05 (0.56)
C14:0	87.50 ± 6.04 (3.18)	61.47 ± 5.21 (2.31)	501.70 ± 45.57 (3.52)	66.93 ± 1.53 (3.67)	729.97 ± 30.56 (5.85)	17.15 ± 0.08 (0.74)
C16:0	432.31 ± 38.20 (15.72)	567.91 ± 40.35 (21.38)	3080.47 ± 80.24 (21.58)	310.91 ± 25.70 (17.03)	4083.80 ± 85.30 (30.11)	623.70 ± 25.30 (26.99)
C18:0	123.49 ± 12.27 (4.49)	169.36 ± 20.10 (6.38)	497.10 ± 38.10 (3.48)	80.20 ± 4.20 (4.39)	1020.95 ± 40.14 (7.53)	85.82 ± 5.36 (3.71)
C20:0	76.05 ± 4.10 (2.77)	27.71 ± 2.16 (1.04)	155.07 ± 17.80 (1.09)	45.50 ± 1.80 (2.49)	99.12 ± 0.80 (0.73)	8.43 ± 0.10 (0.40)
C22:0	93.18 ± 6.43 (3.39)	56.79 ± 1.60 (2.14)	118.03 ± 9.16 (0.83)	30.47 ± 1.05 (1.67)	52.53 ± 0.55 (0.39)	6.80 ± 0.05 (0.30)
C24:0	59.76 ± 2.34 (2.17)	54.98 ± 2.04 (2.07)	131.04 ± 7.55 (0.92)	33.05 ± 0.70 (1.81)	63.44 ± 0.40 (0.47)	5.47 ± 0.10 (0.24)
SFA	989.48 ± 42.55 (35.99)	994.76 ± 50.65 (37.45)	4775.47 ± 96.10 (33.46)	667.81 ± 26.50 (36.58)	6256.54± 110.40 (46.13)	764.39 ± 32.40 (33.12)
C14:1	35.36 ± 4.27 (1.29)	36.71 ± 1.05 (1.38)	110.52 ± 6.30 (0.77)	18.37 ± 0.10 (1.01)	185.36 ± 1.20 (1.37)	89.82 ± 8.10 (3.89)
C16:1	178.11 ± 15.34 (6.48)	344.93 ± 18.30 (12.99)	1677.47 ± 48.57 (11.75)	187.80 ± 0.55 (10.29)	2022.07 ± 40.35 (14.91)	390.28 ± 20.45 (16.76)
c-C18:1n-9	230.46 ± 24.45 (8.38)	131.88 ± 10.46 (4.97)	1177.44 ± 33.10 (8.25)	168.28 ± 1.40 (9.22)	2230.23 ± 38.50 (16.44)	126.88 ± 2.30 (5.49)
C20:1	78.81 ± 5.18 (2.87)	32.55 ± 1.50 (1.23)	284.69 ± 12.04 (1.99)	41.48 ± 0.25 (2.27)	237.89 ± 6.40 (1.75)	17.02 ± 0.06 (0.74)
C21:1	97.69 ± 7.10 (3.55)	98.15 ± 7.90 (3.70)	533.78 ± 28.60 (3.74)	36.49 ± 0.58 (2.00)	302.32 ± 8.25 (2.23)	8.36 ± 0.03 (0.36)
C24:1	82.48 ± 4.20 (3.00)	34.74 ± 1.10 (1.31)	337.39 ± 15.10 (2.36)	31.86 ± 0.20 (1.74)	153.64 ± 2.10 (1.13)	4.10 ± 0.10 (0.20)
MUFA	702.91 ± 34.50 (25.57)	678.97 ± 35.60 (25.56)	4121.30 ± 65.30 (28.88)	484.28 ± 41.80 (26.53)	5131.51 ± 86.55 (37.84)	636.46 ± 50.35 (27.44)
C18:3n-6	51.37 ± 5.53 (1.87)	41.81 ± 2.06 (1.57)	174.66 ± 10.55 (1.22)	24.13 ± 0.08 (1.32)	151.66 ± 1.30 (1.12)	4.10 ± 0.20 (0.20)
C18:2n-6	109.50 ± 9.25 (3.98)	102.56 ± 8.10 (3.86)	1056.66 ± 45.50 (7.40)	102.35 ± 1.10 (5.61)	317.19 ± 4.05 (2.34)	67.76 ± 7.35 (2.93)
C18:3n-3	100.81 ± 10.04 (3.67)	52.39 ± 4.05 (1.97)	351.37 ± 8.35 (2.46)	45.90 ± 0.80 (2.51)	96.10 ± 1.04 (0.71)	26.42 ± 2.24 (1.14)
C20:3n-3	90.61 ± 6.38 (3.30)	54.12 ± 3.70 (2.04)	298.84 ± 10.43 (2.09)	38.23 ± 0.35 (2.09)	60.46 ± 0.30 (0.45)	14.18 ± 0.10 (0.61)
C20:3n-6	55.21 ± 3.55 (2.01)	25.09 ± 0.55 (0.94)	188.87 ± 5.70 (1.32)	22.33 ± 0.15 (1.22)	54.52 ± 0.30 (0.40)	7.25 ± 0.05 (0.31)
C20:2n-6	52.43 ± 4.05 (1.91)	37.87 ± 1.03 (1.43)	239.92 ± 2.75 (1.68)	tr	59.47 ± 0.25 (0.44)	7.85 ± 0.08 (0.34)
C20:4n-6	85.14 ± 6.20 (3.10)	67.35 ± 3.57 (2.54)	585.07 ± 30.20 (4.11)	73.89 ± 0.88 (4.05)	594.73 ± 23.10 (4.39)	67.88 ± 5.30 (2.95)
C20:5n-3	124.25 ± 11.10 (4.52)	109.16 ± 8.05 (4.11)	342.09 ± 10.76 (2.40)	81.54 ± 1.05 (4.47)	148.68 ± 2.06 (1.10)	87.55 ± 8.10 (3.80)
C22:6n-3	339.12 ± 25.15 (12.33)	463.56 ± 30.48 (17.45)	1885.52 ± 68.70 (13.21)	260.27 ± 5.56 (14.26)	634.38 ± 15.30 (4.68)	608.95 ± 35.40 (26.35)
C22:2n-9	48.46 ± 3.07 (1.76)	28.36 ± 1.80 (1.07)	250.07 ± 12.20 (1.75)	23.04 ± 0.30 (1.26)	57.49 ± 0.20 (0.42)	19.50 ± 1.05 (0.85)
PUFA	1056.90 ± 58.10 (38.44)	982.27 ± 55.60 (36.98)	5375.08 ± 98.45 (37.66)	673.54 ± 32.82 (36.89)	2174.72 ± 65.14 (16.04)	911.44 ± 54.10 (39.44)

Results represent mean values ± standard deviation (n = 3); SFA: saturated fatty acids; MUFA: monounsaturated fatty acids; PUFA: polyunsaturated fatty acids; n-3: omega-3; nd: not detected; tr: trace amounts, <0.1%.

**Table 3:** Nutritional quality indexes of 11 fish species from the Black Sea.

	*Spratus* *spratus*	*Engraulis* *encrasih.*	*Trahurus* *mediterr.*	*Alosa* *immaculata*	*Mugil* *cephallus*	*Sarda* *sarda*	*Belone* *belone*	*Pomatomus* *saltatrix*	*Neogobius* *melanost.*	*Mullus* *barbatus*	*Scophthalmus maximus*
n-3, mg.100^−1^ ww	680.58 ± 19.25	2562.23 ± 50.10	1068.81 ± 35.20	2615.30 ± 30.10	558.89 ± 23.45	654.79 ± 20.20	679.23 ± 34.50	2877.83 ± 74.50	425.94 ± 30.10	939.67 ± 63.55	737.14 ± 52.45
n-6, mg.100^−1^ ww	397.12 ± 40.41	817.90 ± 37.40	823.43 ± 30.35	1357.82 ± 90.10	640.28 ± 20.45	402.11 ± 15.33	303.04 ± 17.20	2497.25 ± 90.55	247.60 ± 15.10	1235.05 ± 85.20	174.17 ± 10.55
n-6/n-3	0.58 ± 0.08	0.32 ± 0.03	0.77 ± 0.05	0.52 ± 0.10	1.15 ± 0.15	0.62 ± 0.07	0.45 ± 0.08	0.87 ± 0.11	0.58 ± 0.08	1.31 ± 0.25	0.24 ± 0.02
PUFA/SFA	1.11 ± 0.10	1.04 ± 0.14	1.01 ± 0.07	0.89 ± 0.50	0.95 ± 0.60	1.07 ± 0.57	0.99 ± 0.10	1.13 ± 0.85	1.01 ± 0.10	0.35 ± 0.02	1.19 ± 0.80
DHA/EPA	3.00±	5.34 ± 0.20	2.93 ± 0.30	8.05 ± 1.20	2.77 ± 0.90	2.73 ± 1.20	4.25 ± 2.45	6.52 ± 1.10	3.19 ± 0.80	4.27 ± 0.50	6.96 ± 1.08
AI	0.49 ± 0.02	0.59 ± 0.05	0.58 ± 0.08	0.59 ± 0.05	0.68 ± 0.35	0.49 ± 0.08	0.52 ± 0.05	0.56 ± 0.08	0.55 ± 0.09	1.01 ± 0.32	0.46 ± 0.10
TI	0.23 ± 0.01	0.32 ± 0.03	0.31 ± 0.05	0.38 ± 0.06	0.43 ± 0.14	0.26 ± 0.03	0.32 ± 0.05	0.34 ± 0.03	0.27 ± 0.03	0.97 ± 0.11	0.27 ± 0.05
h/H	1.98 ± 0.60	1.20 ± 0.23	1.85 ± 1.10	1.35 ± 0.45	1.35 ± 0.90	1.91 ± 0.95	1.47 ± 0.40	1.53 ± 0.73	1.94 ± 0.65	0.82 ± 0.09	1.54 ± 0.85

**Table 4:** Total lipids (g/100 g wet weight) and fatty acid composition (mg.100^−1^ g), in the brackets (% of total FAs) of 5 shellfish species from the Black Sea.

	*Chamelea gallina*	*Donax trunculus*	*Mytilus galloprovincialis*	*Rapana venosa*	*Crangon crangon*
Total lipids (g.100^−1^ g ww)	2.26 ± 0.08	1.42 ± 0.06	2.59 ± 0.05	0.81 ± 0.03	1.40 ± 0.05
FA content (mg.100^−1^ g)					
C12:0	nd	nd	nd	0.88 ± 0.68 (0.15)	8.12 ± 0.55 (0.70)
C14:0	17.46 ± 2.48 (0.94)	10.38 ± 0.67 (0.98)	21.45 ± 1.28 (0.98)	15.62 ± 2.03 (2.65)	13.46 ± 0.84 (1.10)
C15:0	3.18 ± 0.33 (0.17)	2.26 ± 0.01 (0.21)	7.32 ± 1.00 (0.34)	5.23 ± 0.59 (0.89)	nd
C16:0	236.41 ± 9.15 (12.68)	132.08 ± 17.73 (12.44)	376.72 ± 19.33 (17.28)	79.30 ± 4.93 (13.44)	317.61 ± 21.55 (27.38)
C17:0	40.45 ± 18.64 (2.17)	5.76 ± 0.25 (0.54)	20.49 ± 2.24 (0.94)	11.49 ± 1.40 (01.95)	3.48 ± 0.23 (0.30)
C18:0	74.05 ± 5.38 (3.97)	73.41 ± 3.07 (6.92)	86.83 ± 2.87 (3.98)	95.96 ± 10.66 (16.26)	49.3 ± 1.72 (4.25)
C20:0	tr	tr	tr	0.65 ± 0.29 (0.11)	8.7 ± 0.16 (0.75)
C21:0	tr	tr	tr	nd	2.3 ± 0.07 (0.20)
C22:0	tr	tr	nd	6.56 ± 1.96 (1.11)	9.98 ± 0.58 (0.86)
C23:0	tr	nd	nd	nd	1.77 ± 0.34 (0.15)
C24:0	tr	tr	tr	7.70 ± 7.72 (1.30)	6.38 ± 0.11 (0.55)
SFA	379.01 ± 31.33 (20.33)	226.16 ± 20.93 (21.30)	555.71 ± 24.92 (25.49)	223.65 ± 22.74 (37.90)	422.8 ± 35.50 (36.49)
C14:1	tr	tr	tr	nd	5.22 ± 0.06 (0.45)
C16:1	52.66 ± 7.61 (2.82)	11.64 ± 0.74 (1.10)	171.21 ± 8.75 (7.85)	8.55 ± 0.69 (1.45)	186.76 ± 10.05 (16.10)
C17:1	tr	tr	nd	2.03 ± 0.59 (0.34)	3.48 ± 0.18(0.30)
c-C18:1n-9	74.44 ± 44.38 (3.99)	51.61 ± 8.81 (4.86)	53.34 ± 63.74 (2.45)	8.67 ± 1.29 (1.47)	92.8 ± 1.23 (8.00)
C20:1	70.09 ± 1.11 (3.76)	40.83 ± 1.64 (3.85)	74.61 ± 3.61 (3.42)	16.18 ± 3.23 (2.74)	8.12 ± 0.24 (0.70)
C22:1	tr	tr	tr	tr	4.64 ± 0.05 (0.40)
C24:1	tr	tr	tr	nd	4.06 ± 0.07 (0.35)
MUFA	201.30 ± 38.69 (10.80)	105.24 ± 10.20 (9.91)	301.61 ± 53.60 (13.83)	35.91 ± 4.83 (6.09)	305.10 ± 48.56(26.30)
C18:2n-6	14.40 ± 1.02 (0.77)	7.34 ± 0.66 (0.69)	25.97 ± 2.32 (1.19)	5.31 ± 0.35 (0.90)	87.35 ± 5.27 (7.53)
C18:3n-6	nd	nd	nd	nd	9.86 ± 0.14 (0.85)
C18:3n-3	8.57 ± 1.04 (0.46)	8.72 ± 2.45 (0.82)	10.17 ± 1.13 (0.47)	1.46 ± 0.18 (0.25)	21.46 ± 2.58 (1.85)
C20:2n-6	52.50 ± 1.41 (2.82)	55.14 ± 3.25 (5.19)	54.56 ± 8.85 (2.50)	39.09 ± 4.24 (6.62)	6.15 ± 0.09 (0.53)
C20:3n-6	nd	nd	19.49 ± 33.76 (0.89)	nd	9.28 ± 0.17 (0.80)
C20:3n-3	6.20 ± 2.08 (0.33)	2.41 ± 1.25 (0.23)	tr	0.77 ± 0.79 (0.13)	9.28 ± 0.06 (0.80)
C20:4n-6	186.91 ± 15.24 (10.02)	89.43 ± 4.96 (8.42)	414.33 ± 20.94 (19.01)	6.57 ± 1.77 (1.11)	44.66 ± 1.48 (3.85)
C22:2n-9	nd	4.00 ± 6.92 (0.38)	nd	nd	3.48 ± 0.10 (0.30)
C20:5n-3	93.67 ± 4.89 (5.02)	46.93 ± 5.83 (4.42)	102.00 ± 4.70 (4.68)	173.70 ± 7.43 (29.44)	69.0 ± 3.24 (5.95)
C22:6n-3	805.62 ± 10.81 (43.21)	444.74 ± 17.27 (41.90)	652.17 ± 28.43 (29.92)	79.36 ± 15.28 (13.45)	171.10 ± 21.55 (14.75)
C22:5n-3	109.19 ± 4.93 (5.86)	61.13 ± 4.05 (5.76)	34.25 ± 8.76 (1.57)	40.88 ± 13.62 (6.93)	nd
C22:4n-3	7.19 ± 1.01 (0.39)	10.29 ± 9.81 (0.97)	7.52 ± 4.21 (0.35)	nd	nd
PUFA	1284.25 ± 13.14 (68.88)	730.13 ± 13.80 (68.78)	1322.73 ± 48.44 (60.67)	330.51 ± 24.73 (56.01)	431.6 ± 26.30 (37.21)

Results represent mean values ± standard deviation (n = 3); SFA: saturated fatty acids; MUFA: monounsaturated fatty acids; PUFA: polyunsaturated fatty acids; n-3: omega-3; nd: not detected; tr: trace amounts.

**Table 5:** Nutritional quality indexes of 5 shellfish species from the Black Sea.

	*Mytilus galloprovincialis*	*Chamelea gallina*	*Donax trunculus*	*Rapana venosa*	*Crangon crangon*
n-3 mg.100^−1^ ww	808.38 ± 34.57	1030.44 ± 29.25	574.22 ± 4.47	296.72 ± 3.18	270.84 ± 4.53
n-6 mg.100^−1^ ww	514.35 ± 23.72	253.81 ± 14.78	155.90 ± 14.06	50.96 ± 5.45	157.52 ± 12.63
n-6/n-3	0.64 ± 0.03	0.25 ± 0.01	0.27 ± 0.03	0.17 ± 0.02	0.57 ± 0.05
PUFA/SFA	2.38 ± 0.14	3.40 ± 0.27	3.25 ± 0.34	1.49 ± 0.25	1.02 ± 0.10
DHA/EPA	6.40 ± 0.27	8.62 ± 0.55	9.60 ± 1.58	0.46 ± 0.10	2.48 ± 0.30
AI	0.28 ± 0.02	0.21 ± 0.01	0.21 ± 0.03	0.37 ± 0.02	0.52 ± 0.05
TI	0.17 ± 0.01	0.10 ± 0.01	0.11 ± 0.01	0.20 ± 0.02	0.36 ± 0.03
h/H	3.17 ± 0.27	4.67 ± 0.32	4.60 ± 0.56	2.91 ± 0.19	1.52 ± 0.20

**Table 6:** Recommended daily serving (500 mg EPA + DHA per day) for Black Sea fish and shellfish.

Species	DHA + EPA, mg.100 g^−1^ ww	Optimum Consumption (g/day)	% of RDI
***Pelagic fish species***			
*Sprattus sprattus*	768.43	65.1	153.7%
*Engraulis encrasicolus ponticus*	2328.7	21.5	465.7%
*Trahurus mediterraneus ponticus*	1035.0	48.3	207.0%
*Alosa immaculata*	2228.9	22.4	445.8%
*Mugil cephallus*	422.6	118.3	84.5%
*Sarda sarda*	463.4	107.9	92.6%
*Belone belone*	572.7	87.3	114.5%
*Pomatomus saltatrix*	2227.6	22.4	445.52%
***Demersal fish species***			
*Neogobius melanostomus*	341.8	146.3	68.4%
*Mullus barbatus*	783.1	63.8	156.62%
*Scophthalmus maximus*	696.5	71.8	139.3%
***Shellfish***			
*Mytilus galloprovincialis*	754.2	66.3	150.8%
*Chamelea gallina*	899.3	55.6	180.0%
*Donax trunculus*	491.7	101.6	98.3%
*Rapana venosa*	253.0	197.6	50.6%
*Crangon crangon*	240.1	208.2	48.0%

## Data Availability

Not applicable.
